# Supplementary figures and images for: Real time structural search of the Protein Data Bank
Source: PLoS Comput Biol. 2020 Jul 8;16(7):e1007970. doi: 10.1371/journal.pcbi.1007970 (PMC7371193; doi:10.1371/journal.pcbi.1007970)

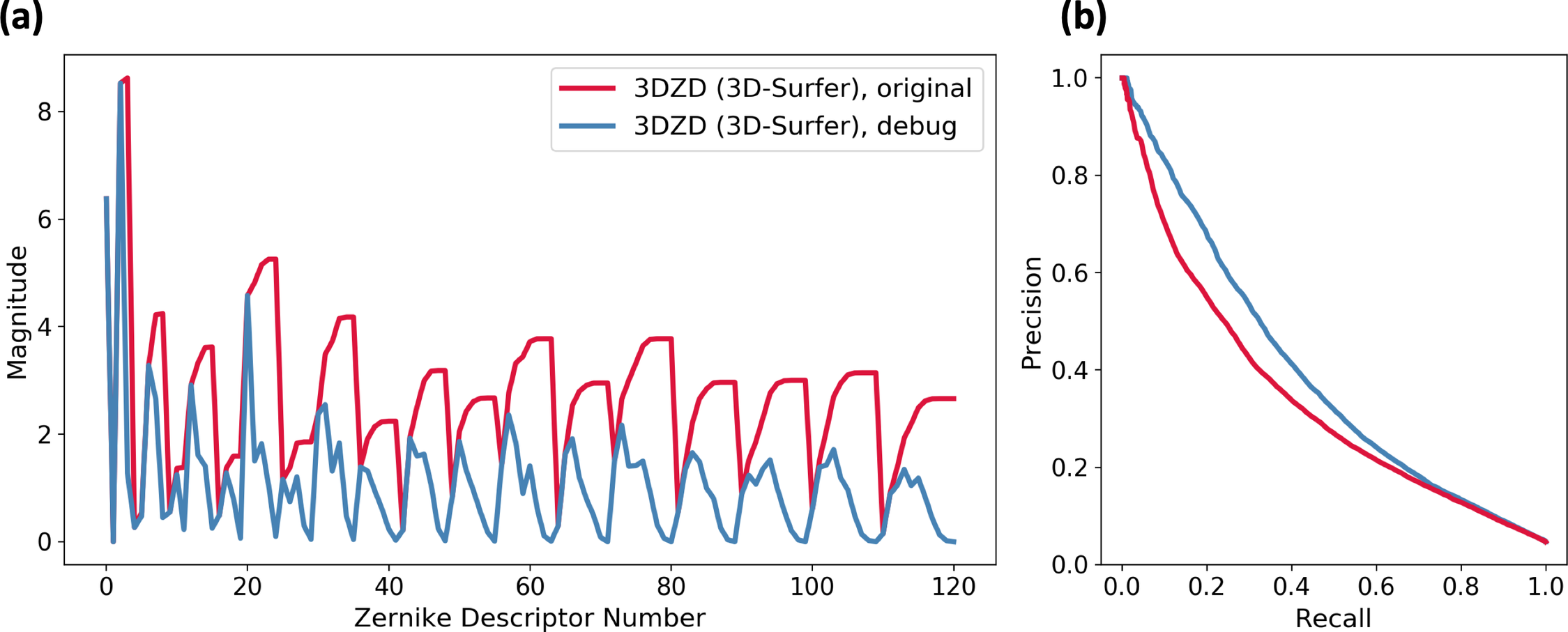

Supplement: S1 Fig — (a) The 3DZD library bug results in characteristic non-decreasing wave pattern (red) of the descriptor, often found in literature. The same descriptor without the bug is shown for comparison in blue. (b) Precision-recall curve using Euclidean distance on the test set improves with the corrected version of the descriptor. (TIF) [file pcbi.1007970.s001.tif]

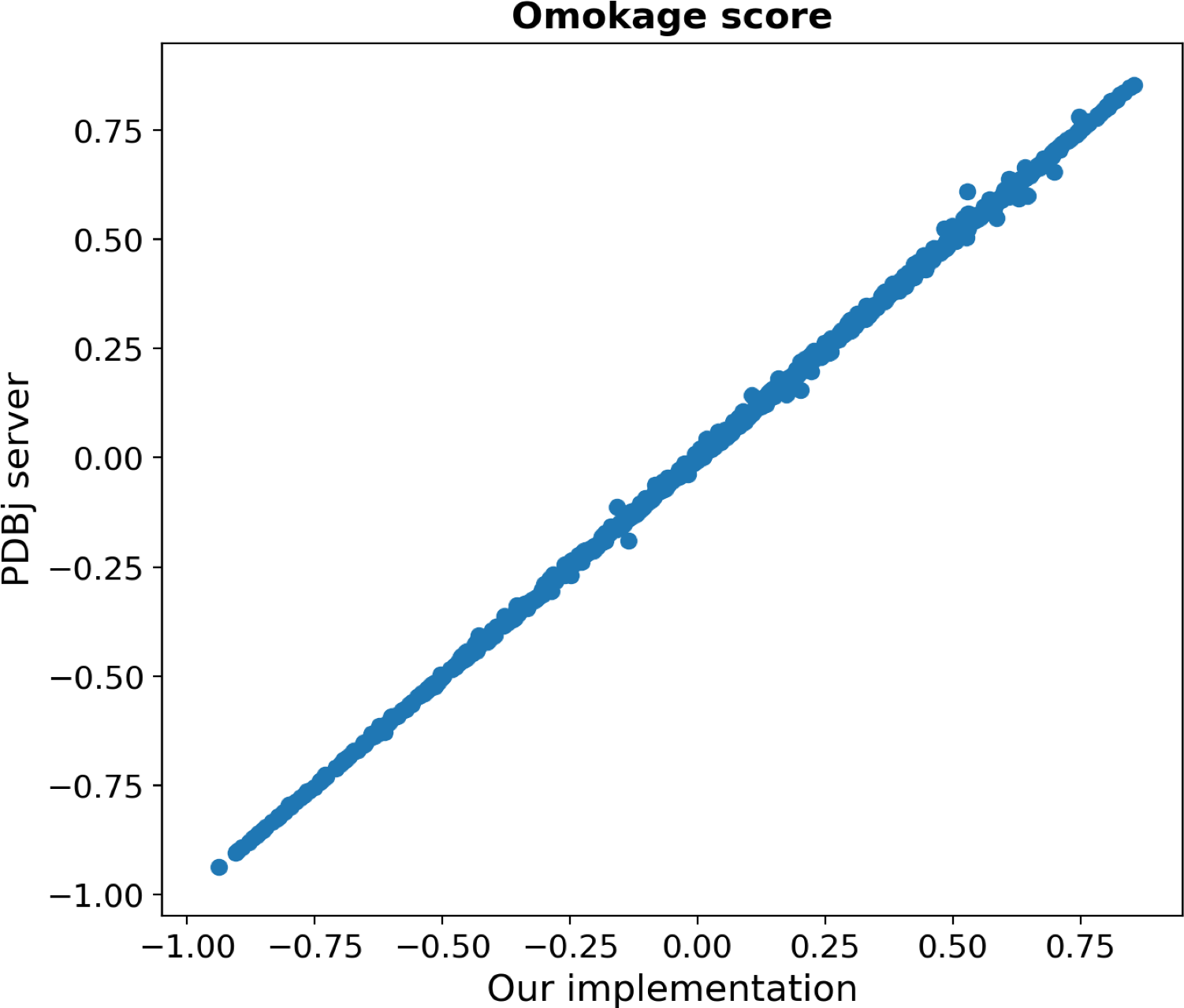

Supplement: S2 Fig — The score calculated by our implementation (axis X) is plotted versus the score obtained from the PDBj server (axis Y) for 1000 random comparisons between structures in PDB. (TIF) [file pcbi.1007970.s002.tif]
