# Supplementary material for: Real time structural search of the Protein Data Bank
Source: PLoS Comput Biol. 2020 Jul 8;16(7):e1007970. doi: 10.1371/journal.pcbi.1007970 (PMC7371193; doi:10.1371/journal.pcbi.1007970)
Supplement: Attachment — Submitted filename: reviewer-comments.pdf [file pcbi.1007970.s003.pdf]

This work describes the development of a distance metric for comparing 3D “electron density maps” using a new normalised form of Zernike moments. The developed metric is applied to the comparison of protein structures by generating coarse-grained electron density maps from structures and searching for similar domains/assemblies in the PDB by comparing their new Zernike moments (CNs) and some geometric descriptors. The work is interesting and complete, and the method for making the Zernike moments rotationally-invariant is an elegant solution. However, expansion and clarification of several areas, particularly in the methods section would improve the readability of the work. Excellent to see that the code is being released open source.

1) Title. The title of the paper could be tweaked to make it more descriptive of the work. Beginning with “preparing for the data deluge” invokes ideas that this is a piece of work describing synchrotron/computational infrastructure — or is a review — and as such does not hint that this is a methodological paper. You could consider changing the title to reflect the content more.

2) Throughout. The authors alternate between using “electron density maps”, “electron density volumes” and “electron density distributions” throughout the work. The use of “electron density maps” is rather confusing, since a reader may initially assume (as I did myself) that this is being applied to experimental map data. Using a generic term like “electron density volume” consistently throughout may reduce any potential confusion. Using a specific (though rather clunky) term like “coarse-grained idealised electron density” would reduce confusion further.

3) Introduction. The use of “data not shown” in the introduction seems unnecessary. Example percentage(s), along with (a) date(s) when this information was extracted from the PDB (and potentially a several-word methodology) does not seem difficult to include. Could then include appropriate graphs in supplementary.

4) Methods Section. The description of the method in the results section is very good and describes the method well in general terms. However, the actual methodology section is very sparse and quite difficult to follow. Adding a few explanatory sentences to guide the reader will prevent the need to e.g. jump between three different pieces of text to figure out the motivation for a particular step. Similarly, all symbols/nomenclature used in equations here should be defined (n,m,l, etc.) even if they are following a derivation from another piece of work, or if they are defined in the results section. Again, even though it is explained in the results section, briefly repeating the motivation for (as an example) setting some Zernike moments to zero (to remove rotational degrees of freedom) would greatly improve readability for those not already familiar with the work/field/nomenclature/notation/etc.

5) Methods Section. The description of the “BioZernike descriptors” section should be expanded/elaborated as it is currently a bit too terse so it is not clear what is being done. “the structure is upscaled”… to what? What is the minimum/maximum sampling that it is being up/down-scaled to? how exactly is a ED-volume scaled to/placed in the unit sphere for calculation of the Zernike moments? (this presumably has a great effect on the calculated moments!).

6) Methods Section. It is not stated clearly why each normalisations with nominally 121 Zernike moments lead to a 946-length vector; the calculation between this should be written explicitly.

7) Methods/Results sections. The authors talk about alternative solutions in several places (either the alternative polynomial solutions or the alternative normalisations) and it is not always clear which they are talking about: e.g. “the absolute values of the alternative solutions are averaged in each case” (methods sections, 2.2). Clarification of language throughout the methods and results sections is essential as it is currently ambiguous.

8) Distance function and benchmarking sections. Will supplementary material containing the set of benchmarking structures and the optimised distance weights be made available with the work?

9) Discussion. The sentence in the discussion “Moreover, this method can be applied directly to the growing number of electric Coulomb potential maps obtained using 3D electron

microscopy” is very unclear. Do the authors mean that you can apply this method to cryo-EM structures, or to the experimental maps? If it is the former, this is trivially true, and the statement should be corrected to be clear they are referring to structures. If the authors are indeed referring to analysis of the experimental maps, then it is not clear that this statement is true, since the coarse-grained map-generation approach in this work has neatly circumvented a major electron-density comparison problem: B-factors (or other disorder effects). (a) It is not clear what analysis the authors have in mind for map-comparison (as the same analyses described in the paper can be performed on the structures directly), and (b) though there are important differences between cryo-EM maps and crystallographic maps, it is not clear why cryo-EM maps are singled out here as being different to crystallographic maps, since both contain sources of disorder that lead to non-zero local B-factors that preclude map-comparison.

10) Figure 2. By what metric is the optimum normalisation order selected? “Normalisation order 4 is selected automatically”. Please clarify.

11) Figure 3. Non-standard alphabet of a-b-c-e-d seems to be used for subfigures. Additionally, some of the labels seem to be misleadingly placed: not sure if arrow from (d) to “Biozernike descriptor” is needed, as this implies the weights are not optimised. Consider tidying/refactoring the flowchart for clarity. Are the lines in (d) from different normalisation really offset from each other so systematically or is this for visualisation? If for visualisation, put them on different y-axes to prevent conclusion.

12) Figure 4. Not colour-blind friendly (even to non-colour-blind people). mix in some dashed/dotted lines to disambiguate similar colours.
