# Supplementary material for: Real time structural search of the Protein Data Bank
Source: PLoS Comput Biol. 2020 Jul 8;16(7):e1007970. doi: 10.1371/journal.pcbi.1007970 (PMC7371193; doi:10.1371/journal.pcbi.1007970)
Supplement: Attachment — Submitted filename: BioZernike review - PLoS CB.pdf [file pcbi.1007970.s004.pdf]

We are grateful to the reviewers for appreciating our work and for the thoroughness of the review. The feedback was very valuable and we think the changes that we have made significantly improved the clarity of the manuscript.

Reviewer #1

This work describes the development of a distance metric for comparing 3D “electron density maps” using a new normalised form of Zernike moments. The developed metric is applied to the comparison of protein structures by generating coarse-grained electron density maps from structures and searching for similar domains/assemblies in the PDB by comparing their new Zernike moments (CNs) and some geometric descriptors. The work is interesting and complete, and the method for making the Zernike moments rotationally-invariant is an elegant solution. However, expansion and clarification of several areas, particularly in the methods section would improve the readability of the work. Excellent to see that the code is being released open source.

Comments

1) Title. The title of the paper could be tweaked to make it more descriptive of the work. Beginning with “preparing for the data deluge” invokes ideas that this is a piece of work describing synchrotron/computational infrastructure — or is a review — and as such does not hint that this is a methodological paper. You could consider changing the title to reflect the content more.

The reviewer raises a good point, the title comes from an older version of the manuscript and in hindsight looks inadequate now. We have changed the title to “Real time structural search of the Protein Data Bank”

2) Throughout. The authors alternate between using “electron density maps”, “electron density volumes” and “electron density distributions” throughout the work. The use of “electron density maps” is rather confusing, since a reader may initially assume (as I did myself) that this is being applied to experimental map data. Using a generic term like “electron density volume” consistently throughout may reduce any potential confusion. Using a specific (though rather clunky) term like “coarse-grained idealised electron density” would reduce confusion further.

An important clarification is that the method we present can work for either experimental volumes or simulated volumes. When in the manuscript we refer to the words “map”, “volume” or “distribution”, we are talking about the mathematical concept of density distribution (an $\mathbb{R}^3$ -> $\mathbb{R}^1$ function). The distribution can be built (“simulated”) from an atomic model (as we do by creating a gaussian distribution around Calpha atoms) or it can come straight from experimental data, though we don’t demonstrate use of experimental data within this work.

We have added a sentence in the last paragraph of the introduction to clarify this point. Moreover, we have standardised the terminology to “electron density volume” whenever appropriate, using “distribution” and “map” only when required by the context.

3) Introduction. The use of “data not shown” in the introduction seems unnecessary. Example percentage(s), along with (a) date(s) when this information was extracted from the PDB (and potentially a several-word methodology) does not seem difficult to include. Could then include appropriate graphs in supplementary.

We have now changed it in the manuscript. Thanks for pointing it out.

4) Methods Section. The description of the method in the results section is very good and describes the method well in general terms. However, the actual methodology section is very sparse and quite difficult to follow. Adding a few explanatory sentences to guide the reader will prevent the need to e.g. jump between three different pieces of text to figure out the motivation for a particular step. Similarly, all symbols/nomenclature used in equations here should be defined (n,m,l, etc.) even if they are following a derivation from another piece of work, or if they are defined in the results section. Again, even though it is explained in the results section, briefly repeating the motivation for (as an example) setting some Zernike moments to zero (to remove rotational degrees of freedom) would greatly improve readability for those not already familiar with the work/field/nomenclature/notation/etc.

We concur that the methods section, while essentially complete, is not easy to follow on its own. We have moved some text from the results section to ease a reader into the manuscript and added a “methods introduction” section with relevant definitions from the cited work.

5) Methods Section. The description of the “BioZernike descriptors” section should be expanded/elaborated as it is currently a bit too terse so it is not clear what is being done. “the structure is upscaled”… to what? What is the minimum/maximum sampling that it is being up/down-scaled to? how exactly is a ED-volume scaled to/placed in the unit sphere for calculation of the Zernike moments? (this presumably has a great effect on the calculated moments!).

We have added more details regarding the volume preparation for the Zernike moments calculation. In addition, we have more formally introduced the alignment descriptor here, as it was implied, but not defined in the manuscript.

6) Methods Section. It is not stated clearly why each normalisations with nominally 121 Zernike moments lead to a 946-length vector; the calculation between this should be written explicitly.

We have now made it clear in manuscript that the difference comes from the m index.

7) Methods/Results sections. The authors talk about alternative solutions in several places (either the alternative polynomial solutions or the alternative normalisations) and it is not always clear which they are talking about: e.g. “the absolute values of the alternative solutions are averaged in each case” (methods sections, 2.2). Clarification of language throughout the methods and results sections is essential as it is currently ambiguous.

We agree that this may be a source of confusion. We have made the terminology more consistent throughout the manuscript. Namely, we talk about “multiple solutions” when referring to the polynomial equations and “alternative normalisations” for the Canterakis norms.

8) Distance function and benchmarking sections. Will supplementary material containing the set of benchmarking structures and the optimised distance weights be made available with the work?

We have made all training and benchmarking data available at https://github.com/rcsb/biozernike-validation . The optimised distance weights, as used in rcsb.org (see new paragraph in section 3.3), are also documented there.

9) Discussion. The sentence in the discussion “Moreover, this method can be applied directly to the growing number of electric Coulomb potential maps obtained using 3D electron microscopy” is very unclear. Do the authors mean that you can apply this method to cryo-EM structures, or to the experimental maps? If it is the former, this is trivially true, and the statement should be corrected to be clear they are referring to structures. If the authors are indeed referring to analysis of the experimental maps, then it is not clear that this statement is true, since the coarse-grained map-generation approach in this work has neatly circumvented a major electron-density comparison problem: B-factors (or other disorder effects). (a) It is not clear what analysis the authors have in mind for map-comparison (as the same analyses described in the paper can be performed on the structures directly), and (b) though there are important differences between cryo-EM maps and crystallographic maps, it is not clear why cryo-EM maps are singled out here as being different to crystallographic maps, since both contain sources of disorder that lead to non-zero local B-factors that preclude map comparison.

First point: we have rephrased to “the growing number of experimental maps obtained from 3D electron microscopy” to clarify what we mean.

Second point: the fact that we show the application of this method to coarse grained simulated maps does not mean that the method is not applicable to experimental maps with higher resolution and potential noise and/or flexibility in the form of B-factors. The expansion to Zernike moments is in fact a very well suited tool for that purpose, because increasing orders describe

increasingly higher resolution features, akin to the familiar Fourier methods. Noise, flexibility and other such issues are naturally handled by the properties of the moments.
We have added a clarification about this point in the “Discussion” section.

10) Figure 2. By what metric is the optimum normalisation order selected? “Normalisation order 4 is selected automatically”. Please clarify.

Thank you for pointing out this unfortunate omission. We have expanded the “BioZernike descriptors” section with the details on the alignment procedure, and updated the figure legend.

11) Figure 3. Non-standard alphabet of a-b-c-e-d seems to be used for subfigures. Additionally, some of the labels seem to be misleadingly placed: not sure if arrow from (d) to “Biozernike descriptor” is needed, as this implies the weights are not optimised. Consider tidying/ refactoring the flowchart for clarity. Are the lines in (d) from different normalisation really offset from each other so systematically or is this for visualisation? If for visualisation, put them on different y-axes to prevent conclusion.

Thanks so much for the feedback on the figure.
1) We have modified the panel labelling to add clarity.
2) The arrow is needed, because it represents the alignment descriptor, which does not involve weight optimization and represents full 3D Zernike moments and the CN-induced rotations. We have added more information about the alignment descriptor into the methods section to clarify the confusion.
3) The different normalisation orders are indeed offset for visualization. We have now clarified it in the legend and removed the y-axis.

12) Figure 4. Not colour-blind friendly (even to non-colour-blind people). mix in some dashed/ dotted lines to disambiguate similar colours.

We have used a color-blind friendly palette for the lines in figure 4 and introduced distinct line styles for the curves based on the BioZernike/3DZD/Omokage approaches.

Data availability

Have all data underlying the figures and results presented in the manuscript been provided?
No. The approach used for generating the dataset is included but seemingly not the list of structures that resulted. list/spreadsheet of optimised zernike/geometric distance metric weights could also be included.

We have made all training and benchmarking data available at https://github.com/rcsb/biozernike-validation . The optimised distance weights, as used in rcsb.org (see new paragraph in section 3.3), are also documented there.

Reviewer #2

The authors present an approach for fast retrieval of similar structures from PDB, using 3D Zernike moments. The proposed approach is an improvement over existing tools and the speed of the method is a big advantage and shows better performance in authors benchmark. The implementation seems robust and is a useful tool for the community. I have a few minor points that will help with user interpretation of results.

Comments

1) How does the approach perform (and rank) in case of partial alignments where only part of the larger protein is matched?

The reviewer brings up a very good point that we did not cover specifically in the manuscript. The method we are introducing in this paper is not well suited to find partial matches and, as is, would rank very poorly. We have now added a paragraph in the "Discussion" section that clarifies that.

2) What are the safe thresholds of the total score to find close and distantly related structures? How much does Zernike descriptors contribute to the total score in general? A discussion would help users.

We have now added a paragraph in Results (subsection 3.3) that explains the thresholds in the search system that is available at the rcsb.org website. Essentially we chose 2 threshold sets based on training against the assemblies dataset. The "strict" set is intended to minimize false negatives (should include close structures only), whilst the "relaxed" set is intended to minimize false positives (should include more distant structures).

3) The method can deal with EM volumes but the current implementation and tests doesn't include these. It is useful to discuss this although I noted that this is mentioned as part of future developments.

We have expanded the discussion of our method's application to EM volumes, listing the practical issues that still need to be addressed. Moreover, we have included a simple example

in the BioZernike library (https://github.com/biocryst/biozernike) showing how to align a PDB coordinate model to an EM map.

Data availability

Have all data underlying the figures and results presented in the manuscript been provided?
No. The benchmark datasets used for tests

We have made all training and benchmarking data available at https://github.com/rcsb/biozernike-validation . The optimised distance weights, as used in rcsb.org (see new paragraph in section 3.3), are also documented there.
